# Long-term impact of elexacaftor/tezacaftor/ivacaftor on small and large airways in people with cystic fibrosis aged ≥6 years: 24-month real-world evidence from the German Cystic Fibrosis Registry

**DOI:** 10.1183/23120541.00813-2025

**Published:** 2026-03-16

**Authors:** Stefanie Dillenhöfer, Katharina Schütz, Manuel Burkhart, Helmut Ellemunter, Matthias Kappler, Sarah Sieber, Lutz Naehrlich, Folke Brinkmann, Sivagurunathan Sutharsan

**Affiliations:** 1 Department of Pediatric Pulmonology, Cystic Fibrosis Center, University Children's Hospital of Ruhr University Bochum at St. Josef-Hospital, Bochum, Germany; 2 Clinic of Paediatric Pneumology, Allergology and Neonatology, Hannover Medical School and Biomedical Research in Endstage and Obstructive Lung Disease Hannover (BREATH), Member of the German Center for Lung Research (DZL), Hannover, Germany; 3 Mukoviszidose Institut gGmbH, Bonn, Germany; 4 Medical University of Innsbruck, Cystic Fibrosis Centre Innsbruck, Innsbruck, Austria; 5 Department of Pediatrics, Dr von Hauner Children's Hospital, University Hospital, LMU Munich, Munich, Germany; 6 STAT-UP Statistical Consulting and Data Science GmbH, Munich, Germany; 7 Department of Pediatrics, Justus-Liebig-University Giessen, Giessen, Germany; 8 Universities of Giessen and Marburg Lung Center, DZL, Giessen, Germany; 9 Department of Pediatric Pneumology and Allergology, The University of Lübeck, University Medical Center Schleswig-Holstein, Campus Centrum Lübeck, Member of Airway Research Center North of the DZL, Lübeck, Germany; 10 Department of Pulmonary Medicine, University Hospital Essen – Ruhrlandklinik, Adult Cystic Fibrosis Center, University of Duisburg-Essen, Essen, Germany; 11 S. Dillenhöfer and K. Schütz share first authorship; 12 F. Brinkmann and S. Sutharsan share senior authorship; 13 For a list of the members of the German Cystic Fibrosis Registry of the Mukoviszidose e.V. and participating cystic fibrosis sites see the supplementary material

## Abstract

**Background:**

While studies have consistently reported improvements in lung function during elexacaftor/tezacaftor/ivacaftor (ETI) therapy for people with cystic fibrosis (pwCF), individual response predictors remain poorly understood and there is limited real-world data relating to children aged 6–11 years.

**Methods:**

Lung function data from the German CF Registry 2020–2024 were analysed before and up to 24 months after ETI therapy initiation in adults, adolescents and children aged ≥6 years. Data were stratified for percent predicted forced expiratory volume in 1 s (ppFEV_1_) increase during therapy (<5% *versus* >5%), underlying mutation and pre-treatment disease severity.

**Findings:**

Data from 2375 pwCF were analysed. After starting ETI, ppFEV_1_ increased most in adolescents with baseline ppFEV_1_ >40–60% (median increase 16.8–20.2%); the corresponding increase in adults with the same initial lung function was 11.2–11.5%. After an initial increase, ppFEV_1_ remained stable for 24 months in all age groups. No predictors for greater improvement (>5% ppFEV_1_ gain) were identified but pwCF with normal ppFEV_1_ before ETI therapy had smaller increases during treatment. Forced mid-expiratory flow at 25–75% of forced vital capacity increased to a similar extent in children and adolescents (+13%) and adults (+9.5%).

**Interpretation:**

Our real-world data showed significant improvements in lung function, including large and small airways, during 24 months’ ETI therapy in pwCF aged ≥6 years. Notable improvements, particularly in small airways, were observed even in children and adolescents with normal ppFEV_1_ before ETI. These findings underscore the importance of early ETI therapy initiation to prevent irreversible lung damage.

## Shareable abstract

**This real-world study used data from the German CF Registry and provides novel insights into the effects of 24 months’ ETI therapy on large and small airway lung function (FEF_25–75%_) improvement in children aged ≥6 years, adolescent and adult pwCF** https://bit.ly/4gkhKlm

## Introduction

The triple cystic fibrosis transmembrane conductance regulator (CFTR) modulator therapy elexacaftor/tezacaftor/ivacaftor (ETI) in combination with ivacaftor (IVA) was introduced in Germany in August 2020 for people with cystic fibrosis (pwCF) aged ≥12 years, in June 2021 for pwCF aged ≥6 years and in November 2023 for pwCF aged ≥2 years. This treatment represented a significant advancement in therapy options for pwCF who have at least one copy of the *F508del* allele and is currently available for >85% of pwCF in Europe and Northern America.

Clinical trials and observational studies have reported substantial benefits of ETI therapy, including improvements in lung function (percent predicted forced expiratory volume in 1 s (ppFEV_1_)), body mass index (BMI), exacerbation frequency, respiratory symptoms and decreased sweat chloride levels for children aged ≥6 years and adults [1–8]. However, determinants of response to ETI therapy are not yet defined and most published data relates to effects within the first year of therapy. Large, multicentre, real-world studies on the effects of therapy beyond 1 year are scarce and primarily focus on adults [9]. An interim analysis at week 144 during the open-label extension of the pivotal study showed that ETI was effective and safe for pwCF aged ≥12 years [10]. However, real-world data on long-term lung function improvement based on disease severity before ETI initiation, especially in children aged 6–11 years, remains limited.

In addition to changes in ppFEV_1_, small airway obstruction is a hallmark of lung disease in pwCF. During ETI therapy, this has primarily been investigated using the lung clearance index (LCI) *via* multiple breath washout (MBW) [3, 11, 12]. Mid-expiratory flow (forced expiratory flow at 25–75% of forced vital capacity (FEF_25–75%_)), a parameter derived from spirometry, also reflects small airway function but its potential to evaluate small airway improvements during ETI therapy has not yet been thoroughly explored. Furthermore, the impact of the underlying CFTR gene mutation on the response to treatment with ETI remains poorly understood and might potentially influence any lung function response [13].

This real-world study used data from the German CF Registry to determine the effects of ETI therapy on lung function in pwCF aged ≥6 years over a 24-month period, with a focus on small airways (using FEF_25–75%_) and in subgroups based on response to therapy (<5% *versus* ≥5% increase in ppFEV_1_). Effects in children, adolescents and adults were evaluated separately and we evaluated the effects of ETI therapy based on baseline lung function and CFTR mutation to provide a comprehensive understanding of the impact of ETI therapy in different individuals.

## Methods

### Study design, population and treatment

This real-world multicentre, post-approval observational study used encounter-based documentation from the German CF Registry over the period from 1 January 2020 to 25 September 2024. The German CF Registry includes annual data for approximately 80% of all pwCF in Germany (7587 pwCF across 85 centres in 2023) [14]. Encounter-based documentation, established at 67 centres, accounts for 90% of these patients. Clinic visits occur every 3–6 months at participating centres, with final data entered into the Registry.

From this dataset, all pwCF aged ≥6 years who were eligible for ETI and had an ETI therapy duration of ≥24 months were included. ETI therapy with an interruption of up to 90 days was considered continuous therapy. If ETI therapy was restarted after a break of >90 days, only the first period of therapy was included into analysis. Individuals with a history of transplantation (any organ) were excluded. To ensure data reliability, we included patients with consistent data recorded at least every 6 months throughout the study period.

The registry was approved by the Ethics Committee of the Justus-Liebig-University, Gießen, Germany (AZ24/19). Written informed consent was obtained from all participants or their legal guardians. The German CF Registry has a controlled system with “audits” to achieve a high-quality standard of data [15]. The results refer to the annual aggregation in terms of accuracy and show a high degree of consensus.

### Study parameters

Spirometry parameters (ppFEV_1_, FEF_25–75%_) were measured according to American Thoracic Society/European Respiratory Society criteria and analysed using Global Lung Function Initiative equations for Caucasians [12]. We extracted data on demographics, ppFEV_1_, FEF_25–75%_, BMI, pulmonary exacerbations, presence of *Pseudomonas aeruginosa* and CFTR mutations within the 6 months before starting ETI (baseline) and every 6 months during 24 months of ETI therapy. If there was more than one measurement/observation in a 6-month period, then the median of all values was used.

Data were stratified according by age at the start of ETI therapy (children (age ≥6–11 years), adolescents (age ≥12–17 years), adults (age ≥18 years)), pre-treatment with non-ETI CFTR modulators, baseline disease severity (normal (ppFEV_1_ >80%), impaired (ppFEV_1_ >60–80%), moderately impaired (ppFEV_1_ >40–60%) or severely impaired (ppFEV_1_ ≤40%)), change in FEF_25–75%_ referred to baseline ppFEV_1_ and type of mutation (*F508del* homozygous (F/F), *F508del* heterozygous and minimal function (F/MF)), *F508del* heterozygous and residual function (F/RF), *F508del* heterozygous and gating mutation (F/G) and *F508del* heterozygous and other mutation (F/O)). Participants were also classified into one of two groups based on whether they had a <5% or ≥5% increase from baseline in ppFEV_1_ during ETI therapy.

The BMI was reported as *z*-scores for children and adolescents [16] and absolute values in kg·m^–2^ for adults. A pulmonary exacerbation was defined as the need for additional oral or intravenous antibiotic treatment. At least two of the following signs or symptoms must be present to meet the criteria for exacerbation: change in sputum volume or colour; increased cough; increased malaise, fatigue or lethargy; anorexia or weight loss; ≥10% decrease in pulmonary function; radiographic changes; and/or increased dyspnoea [17]. All antibiotic treatments, including those in addition to chronic antibiotic therapy, are explicitly requested and documented by the German CF Registry. In a drop-down menu, the reason for antibiotic therapy can be selected from a predefined list of options. One of this option is “pulmonary exacerbation” as defined above. Only antibiotic treatments specifically prescribed for pulmonary exacerbation were included in the documentation process and subsequent analysis.

### Statistical analysis

Statistical analysis was performed using R version 4.3.2. Baseline demographics and clinical characteristics are presented descriptively using median (interquartile range (IQR)) values or proportions. Because examinations of pwCF occurred on different dates and at different intervals within the registry, consistent time periods were formed for analysis. Hence, ppFEV_1_ values were calculated as the individual median for each 6-month period in the year before and the 2 years after ETI therapy initiation. Baseline values were obtained from the last 6 months before the start of treatment. The first 4 weeks of ETI therapy were not included in the considered time intervals, so that the first 6-month evaluation period started 4 weeks after therapy initiation and ended 6 months plus 4 weeks later. For ppFEV_1_, individual differences between baseline and the 6-month periods during ETI therapy were compared using Wilcoxon signed-rank tests for non-normally distributed data. Comparisons of numeric measures between groups were performed using Wilcoxon rank sum tests for independent samples. The normality assumption for differences and within each group was evaluated using normal quantile–quantile plots. Chi-square tests were used to assess the association between two categorical variables, but the Fisher exact test was used if sample sizes were too small. All p-values calculated in the study were adjusted using the Holm method.

## Results

### Study population and follow-up

**FIGURE 1:**
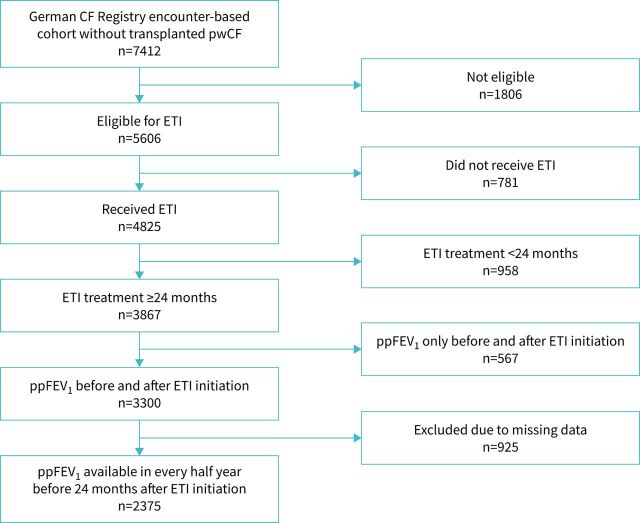
Study flowchart. CF: cystic fibrosis; ETI: elexacaftor/tezacaftor/ivacaftor; ppFEV_1_: percent predicted forced expiratory volume in 1 s; pwCF: people with cystic fibrosis.

Of 7822 pwCF in the German CF Registry encounter-based cohort, 4825 received ETI, 3300 had data from before and during 24 months of ETI therapy, and 2375 (344 children, 527 adolescents, 1504 adults) had spirometric data for ppFEV_1_ recorded at least every 6 months and were included in this analysis (figure 1). The study population had a median age of 23.1 years and 49.3% were female (table 1).

**TABLE 1:** Baseline demographic and clinical characteristics of the study population

Characteristics	Participants (n=2375)
**Age, years**	
Median (IQR)	23.1 (14.3–33.3)
Range	6.0–74.9
**Male sex, n (%)**	1204 (50.7)
**Body mass index, kg·m^−2^**	
n	2369
Median (IQR)	19.8 (17.5–22.2)
Range	12.0–36.5
**Mutation, n (%)**	
*F508del* homozygous	1326 (55.8)
*F508del* heterozygous: minimal function	817 (34.4)
*F508del* heterozygous: residual function	83 (3.5)
*F508del* heterozygous: gating	44 (1.9)
*F508del* heterozygous: other	105 (4.4)
**Exacerbations, n (%)**	628 (26.4)
***Pseudomonas aeruginosa*, n (%)**	948 (39.9)

IQR: interquartile range.

### Lung function

At baseline median ppFEV_1_ was 72.0% (IQR 49.5–89.0%). During the first 6 months of ETI therapy, the individual median ppFEV_1_ had increased by 9.0% after 6 months and remained stable up to 24 months; there was no significant change in median ppFEV_1_ between the first and second years of ETI therapy.

**FIGURE 2:**
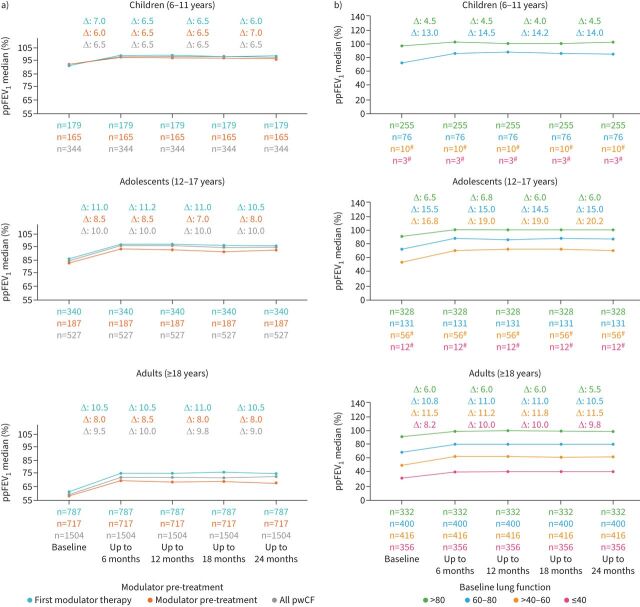
Median change in percent predicted forced expiratory volume in 1 s (ppFEV_1_) from the 6 months before to four 6-month periods after elexacaftor/tezacaftor/ivacaftor (ETI) initiation by aged group and in subgroups based on a) previous modulator therapy or b) baseline ppFEV_1_. Changes (Δ) represent the median of individual differences between the 6-month (half-year) period preceding treatment and the corresponding 6-month periods after treatment initiation for participants with ppFEV_1_ values from the 6-month period before therapy initiation to the fourth 6-month period after starting treatment. pwCF: people with cystic fibrosis. ^#^: Data are not shown if subgroup sample size was <15.

Median ppFEV_1_ at baseline was highest in children (92.0%), who showed a median improvement of 6.5% at 6 months after ETI initiation (figure 2 and table S1). In adolescents, median ppFEV_1_ was 85.0% at baseline and had increased by 10.0% after 6 months (figure 2 and table S1). Adults had the lowest baseline ppFEV_1_ (59.5%) and showed a median improvement of 9.5% at 6 months (figure 2 and table S1).

Across all age groups, increases in ppFEV_1_ during ETI therapy were greater in pwCF with more impaired baseline lung function and in those with impaired lung function at baseline.

Improvements of ppFEV_1_ were higher in children and adolescents with impaired ppFEV_1_ prior ETI compared to adults (figure 2b). When baseline lung function was impaired or moderately impaired, improvements in ppFEV_1_ over 24 months were 15.0% and 20.2%, respectively, in adolescents and 10.5% and 11.5%, respectively, in adults (p<0.001) (table S1). Improvements in ppFEV_1_ during ETI therapy were significantly greater in those who had not *versus* had been pretreated with other CFTR modulators (p<0.001) (figure 2 and table S1).

**FIGURE 3:**
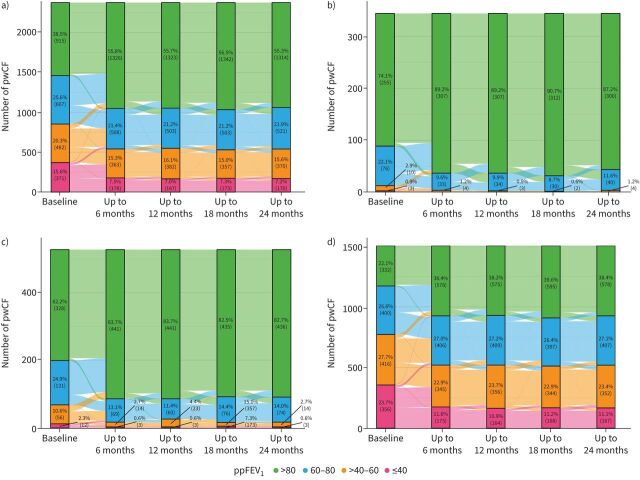
Sankey diagram showing changes in percent predicted forced expiratory volume in 1 s (ppFEV_1_) from the 6 months before to four 6-month periods after initiation of elexacaftor/tezacaftor/ivacaftor for a) all people with cystic fibrosis (pwCF), b) children, c) adolescents and d) adults.

The proportion of pwCF who had normal lung function increased significantly during ETI therapy, from 74.1% to 87.2% in children, from 62.2% to 82.7% in adolescents and from 22.1% to 38.4% in adults (figure 3).

Stratifying ppFEV_1_ improvement by mutation type, pwCF with F/F or F/MF mutations showed significantly greater ppFEV_1_ gains than those with F/RF or F/G mutations (figure S1 and table S1). Adolescents with F/MF or F/F mutations showed the greatest improvements in ppFEV_1_ during ETI therapy. Individuals naive to modulator therapy had greater increases in ppFEV_1_ than those previously treated with CFTR modulator. At ETI initiation, 71.2% of pwCF with an F/F mutation were already on modulator therapy, whereas 97.7% of adults with an F/MF mutation had no prior CFTR modulator treatment (figure S2 and table S1); the number of children and adolescents with F/RF or F/GF mutations was too small to be evaluated. All pwCF with an F/MF mutation showed a greater increase in ppFEV_1_ during the initial months of ETI therapy compared to those with an F/F mutation, but this difference was not sustained after 24 months of treatment.

### Predictors of lung function response

Overall, pwCF who showed a <5% improvement in ppFEV_1_ during ETI therapy had significantly higher baseline ppFEV_1_ (86.0%) compared to those with ≥5% improvement (68.5%, p<0.001) (table S2). This effect is evident in all age-groups: 97.8% *versus* 89.0% baseline ppFEV_1_ in children (p<0.001), 93.8% *versus* 84.0% baseline ppFEV_1_ in adolescents (p<0.001) and 65.0% *versus* 58.0% baseline ppFEV_1_ in adults (p<0.001) (table S3). Age, sex and BMI did not differ significantly between the groups of <5 *versus* ≥5% improvement in ppFEV_1_ (table S2). However, adults with <5% ppFEV_1_ improvement had a higher proportion of CFTR modulator pretreatment (56.6% *versus* 45.4%, p=0.005) (table S3). Prior to ETI therapy initiation, the amount of pulmonary exacerbation in children and adults was significantly lower in those with a <5% *versus* ≥5% ppFEV_1_ improvement (table S3).

### Small airway function

**FIGURE 4:**
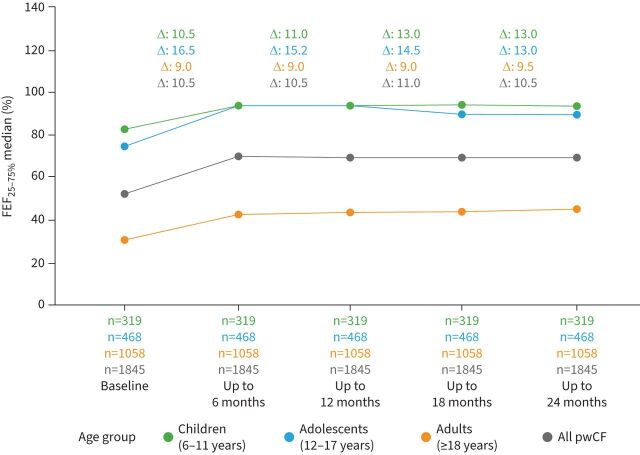
Median change in percent predicted forced mid-expiratory flow at 25–75% of forced vital capacity (FEF_25–75%_) from the 6 months before to four 6-month periods after elexacaftor/tezacaftor/ivacaftor (ETI) initiation by age group. Changes (Δ) represent the median of individual differences between the 6-month (half-year) period preceding treatment and the respective 6-month period after treatment initiation. pwCF: people with cystic fibrosis.

Overall, percent predicted FEF_25–75%_ improved by 10.5% during ETI therapy, with the greatest increase seen in adolescents (16.5%) (figure 4). Similar to change in ppFEV_1_, improvements in percent predicted FEF_25–75%_ were greater in those with impaired baseline lung function than in pwCF with normal baseline lung function; adults with severe baseline impairment had the smallest increase in percent predicted FEF_25–75%_ during treatment with ETI (figure S3 and table S1).

### Exacerbations

All subgroups showed a reduction in the pulmonary exacerbation rate after ETI initiation, except children with a <5% improvement in ppFEV_1_ during therapy (table S3). Exacerbation rates during ETI therapy were highest in children (28.3% and 28.8% in the subgroups with <5% and ≥5% ppFEV_1_ improvement, respectively), intermediate in adults (23.4% and 23.4% in the subgroups with <5% and ≥5% ppFEV_1_ improvement, respectively) and lowest in adolescents (15.3% and 18.3% in the subgroups with <5% and ≥5% ppFEV_1_ improvement, respectively) (table S3).

## Discussion

This real-world study found significant improvements in clinical outcomes, including increased ppFEV_1_, improved small airway function (FEF_25–75%_), higher BMI and a lower rate of pulmonary exacerbations across all age and disease severity groups in a cohort of 2375 pwCF. An important observation is the absence of the typical annual decline in ppFEV1 (1–2%) reported in the pre-CFTR modulator era [18, 19]. Instead, we observed a small variability of 0.5–1% in ppFEV_1_ over 24 months across all age groups. Further studies are needed to evaluate if ETI effectively halts lung function deterioration.

Real-world evidence is essential for understanding the long-term effects of ETI because it includes patients who are often excluded from clinical trials. Our findings underscore the value of registry-based data in capturing outcomes across a broad and unselected patient population, providing insights into the long-term impacts of ETI on pwCF. For rare diseases like CF, where clinical trials have inherent limitations, such data are indispensable for evaluating new treatments and informing clinical practice [20, 21].

The improvements in lung function, nutritional status and pulmonary exacerbation frequency in our real-world cohort align with findings from randomised clinical trials evaluating ETI in pwCF [1, 3, 6, 22–24]. The consistency between our real-world data analysis and results from these trials underscores the reliability and robustness of the registry data and reaffirms the significance of real-world evidence for validating therapeutic outcomes [6].

To date, real-world observational data in pwCF age 6–11 years are mostly limited to 3 months [5, 25]. However, an open-label clinical trial showed comparable data for ppFEV_1_ in a small cohort of 64 children aged 6–11 years during 94 weeks of ETI therapy [7]. Our study extends these findings over a longer time period by providing real-world evidence over 24 months.

Overall, we saw substantial improvements in ppFEV_1_ and FEF_25–75%_ within 6 months after initiation of ETI, which remained stable for 24 months in all age and disease severity groups. Children had the highest ppFEV_1_ at baseline and the median improvement during ETI therapy was 6.5%, smaller than the median ppFEV_1_ improvements seen in adolescents (10.0%) and adults (9.5%). The vast majority of children achieved normal lung function during ETI therapy (figure 4). However, the small number of children with severe baseline lung function impairment precluded detailed analysis of this subgroup, highlighting a need for future research [26].

Adolescents with impaired and moderate impaired baseline lung function showed the greatest improvements in ppFEV_1_ during ETI therapy (15.0% and 20.2%, respectively). In contrast, adults with similar levels of baseline impairment showed smaller gains, likely due to chronic, irreversible lung damage. This observed difference highlights the need for early intervention to maximise the therapeutic benefits of ETI. These findings are consistent with previous real-world studies across different countries, which similarly reported stable long-term lung function during ETI therapy, particularly in younger age groups, with ppFEV_1_ increases ranging from 6.5% to 10.0% [8–10, 27, 28]. To date, large multicentre observational studies on long-term effects of ETI are scarce highlighting the importance of our data. As expected, pwCF pre-treated with any modulator prior ETI initiation showed lower ppFEV_1_ increase after ETI initiation and slightly lower ppFEV_1_ values at all, compared to those who received first modulator therapy. It is likely that a previous modulator therapy has already shown an effect on ppFEV_1_ improvement. Additionally, mainly pwCF with more advanced lung disease as reflects by a lower ppFEV_1_ at all were the first to start modulator therapy in the pre-ETI era.

During ETI therapy, approximately 22% of pwCF had a <5% improvement in ppFEV_1_, including 35% of children, 22% of adolescents and 20% of adults. The higher proportion of children in this category can be attributed to the better baseline lung function observed in children, which leaves less room for improvement. This also underscores the need for age-specific definitions of ETI response to account for the differences in baseline lung function and potential for improvement.

Individuals with a <5% *versus* ≥5% improvement in ppFEV_1_ after 2 years of ETI therapy had significantly higher baseline ppFEV_1_ and FEF_25–75%_, fewer pulmonary exacerbations, and a lower prevalence of *P. aeruginosa* infection. Notably, pwCF who had a <5% increase in ppFEV_1_ during ETI therapy showed no measurable improvement in small airway function. Again, this may have been a consequence of better baseline pulmonary function, limiting the potential for further functional gains.

Our data on changes in small airway function obtained using FEF_25–75%_ are consistent with previous studies that assessed small airway changes using the LCI [2, 6] *via* MBW [3, 11, 29]. Real-world data especially in children on small-airway improvement are scarce. Daccò *et al.* [28] also showed a decrease of LCI in pwCF aged 6–11 years. Due to the limited standardised implementation of MBW in Germany, LCI data were not available from the German CF Registry reflecting the situation in other CF registry-based real-world analysis [30]. However, FEF_25–75%_, a parameter derived from spirometry, offers a practical and accessible alternative for evaluating small airway function, despite its inherent variability and high scatter range.

Across all age groups, BMI consistently increased during 24 months of ETI therapy, regardless of lung function changes. This highlights the broader systemic benefits of ETI beyond respiratory improvements.

Individuals with F/F or F/MF mutations showed the greatest improvements in ppFEV_1_ during ETI therapy. Of these, more than two-thirds of pwCF with F/F mutations were pretreated with modulators whereas the F/MF group were predominantly modulator-naïve, which could potentially explain the similar ppFEV_1_ increases. These findings align with clinical trial data for pwCF aged >6 years [8, 10]. Within the adult subgroup, those with gating mutations showed the highest baseline ppFEV_1_, reflecting prior modulator therapy use (figure S2). To our knowledge, this is the first comprehensive real-world analysis of long-term ppFEV_1_ outcomes stratified by genetic mutation in a cohort of pwCF. No clear gene–dose effect of ETI has been identified to date [13]. Our study has several limitations. First, half of the registry population was excluded due to missing data, discontinuous documentation or age below the inclusion threshold. Nevertheless, the larger centres, in particular, enter their patient data into the German CF Registry and take part in a benchmarking exchange. This is why our dataset of 2400 pwCF is representative for Germany. Initially, we calculated our data in a larger cohort including all pwCF and discontinuous documentation. We did not see significant differences in baseline demographics, but because of a high amount of missing data we decided to include only data of pwCF and continuous documentation. Second, the median of lung function was chosen within a 6-month period. This carries the risk that an exacerbation may bias the result. However, we decided against the use of only the best value in order to depict lung function more realistically as an individual median. Given that patients typically attend visits every 3 months, one to two lung function measurements per 6-month period are included per individual. As exacerbation rates decline after ETI introduction, the statistical influence should not be decisive.

In conclusion, this study presents comprehensive real-world data on the long-term effects of ETI therapy, including improvements in lung function over 24 months in children aged >6 years, adolescents and adults. It highlights the impact of ETI therapy on both large and small airways, (as reflected by ppFEV_1_ and FEF_25–75%_, respectively). Notable improvements were observed even in children and adolescents with normal ppFEV_1_ before ETI. Benefits were consistent across subgroups based on age, sex and BMI, but data suggest that response to ETI may be smaller in adults who have previously received CFTR modulator therapy and in children and adults with lower rate of pulmonary exacerbations. Along with the need to initiate ETI therapy early to prevent irreversible lung damage, our data highlight the importance of personalised treatment strategies to optimise therapeutic outcomes, as suggested previously [31].

## Data Availability

Data that underline the results reported in this article and the individual participant data will not be shared.

## Supplementary material

**Please note:** supplementary material is not edited by the Editorial Office, and is uploaded as it has been supplied by the author. Supplementary material 00813-2025.SUPPLEMENT
