# “Long-term impact of elexacaftor/tezacaftor/ivacaftor on small and large airways in people with cystic fibrosis aged ≥6 years: 24-month real-world evidence from the German Cystic Fibrosis Registry” Stefanie Dillenhöfer, Katharina Schütz, Manuel Burkhart, Helmut Ellemunter, Matthias Kappler, Sarah Sieber, Lutz Naehrlich, Folke Brinkmann and Sivagurunathan Sutharsan on behalf of the German CF Registry of the Mukoviszidose e.V. and participating cystic fibrosis sites. *ERJ Open Res* 2026; 12: 00813-2025.

**DOI:** 10.1183/23120541.50813-2025

**Published:** 2026-07-13

## Abstract

This article was originally published with an error in the author list regarding shared joint authorships. S. Dillenhöfer and K. Schütz share first authorship, and F. Brinkmann and S. Sutharsan are joint senior authors. This has been corrected in the article itself. We apologise for this error.

This article was originally published with an error in the author list regarding shared joint authorships. S. Dillenhöfer and K. Schütz share first authorship, and F. Brinkmann and S. Sutharsan are joint senior authors. This has been corrected in the article itself. We apologise for this error.

