# Supplementary material for: Long-term impact of elexacaftor/tezacaftor/ivacaftor on small and large airways in people with cystic fibrosis aged ≥6 years: 24-month real-world evidence from the German Cystic Fibrosis Registry
Source: ERJ Open Res. 2026 Mar 16;12(2):00813-2025. doi: 10.1183/23120541.00813-2025 (PMC12991016; doi:10.1183/23120541.00813-2025)
Supplement: Supplementary file 1 [file 00813-2025.SUPPLEMENT.pdf]

SUPPLEMENTARY MATERIAL

Long-term impact of elexacaftor/tezacaftor/ivacaftor on small and large airways in pwCF aged ≥6 years: 24-month real-world evidence from the German CF Registry

Stefanie Dillenhoefer[1#], Katharina Schütz[2#], Manuel Burkhart[3], Helmut Ellemunter[4], Matthias Kappler[5], Sarah Sieber[6], Lutz Naehrlich[7,8], Folke Brinkmann[9#], Sivagurunathan Sutharsan[10#] on behalf of the German CF Registry of the Mukoviszidose e.V. and participating CF sites

[1]Department of Pediatric Pulmonology, Cystic Fibrosis Center, University Children's Hospital of Ruhr University Bochum at St. Josef-Hospital, Bochum, Germany

[2]Clinic of Paediatric Pneumology, Allergology and Neonatology, Hannover Medical School, Hannover, Germany; Biomedical Research in Endstage and Obstructive Lung Disease Hannover (BREATH), Member of the German Center for Lung Research (DZL), Germany.

[3]Mukoviszidose Institut gGmbH (MI), Bonn, Germany

[4]Medical University of Innsbruck, Cystic Fibrosis Centre Innsbruck, Innsbruck, Austria

[5]Department of Pediatrics, Dr. von Hauner Children's Hospital, University Hospital, LMU Munich, Germany

[6]STAT-UP Statistical Consulting & Data Science GmbH, Munich, Germany

[7]Department of Pediatrics, Justus-Liebig-University Giessen, Giessen, Germany

[8]Universities of Giessen and Marburg Lung Center (UGMLC), German Center for Lung Research (DZL), Giessen, Germany

[9]Department of Pediatric Pneumology & Allergology, The University of Lübeck, University Medical Center Schleswig-Holstein, Campus Centrum Lübeck, Member of Airway Research Center North (ARCN) of the German Center of Lung Research (DZL), Lübeck, Germany

[10]Department of Pulmonary Medicine, University Hospital Essen - Ruhrlandklinik, Adult Cystic Fibrosis Center, University of Duisburg-Essen, Essen, Germany

Members of the German Cystic Fibrosis Registry of the Mukoviszidose e.V. and participating CF sites in 2023

Title	Surname	Last Name	Department	Hospital Name/Faculty
Dr.	Ines	Adams	Kinderklinik	Otto-von-Guericke Universität Magdeburg
PD Dr.	Andreas	Artlich	Klinik für Kinder und Jugendliche	Oberschwabenklinik (OSK) gGmbH, Ravensburg
Prof. Dr.	Manfred	Ballmann	Kinder- und Jugendklinik Pneumologie und Allergologie	Universitätsmedizin Rostock
Prof. Dr.	Joachim	Bargon	Klinik für Pneumologie	Frankfurter Rotkreuz-Kliniken e.V., Frankfurt

Dr.	Monika	Bauck	Klinik für Kinder- und Jugendmedizin II Pädiatrische Pneumologie	Phillipps Universität Marburg
Dr.	Monika	Bauck	Pädiatrische Hämatologie und Onkologie, Psychosomatik und Systemerkrankungen	Klinikum Kassel
Dr.	Stefan	Blaas	Zentrum für Pneumologie	Klinik Donaustauf
Dr.	Ingrid	Bobis	4. Medizinische Klinik Christiane Herzog Zentrum Nord	Städtisches Klinikum Kiel
PD Dr.	Sebastian	Bode	Klinik für Kinder- und Jugendmedizin	Universitätsklinikum Ulm
Prof. Dr.	Folke	Brinkmann	Campus Lübeck Klinik für Kinder- und Jugendmedizin	Universitätsklinikum Schleswig Holstein, Lübeck
Dr.	Heike	Buntrock-Döpke	Kinder- und Jugendmedizin	Klinik St. Hedwig, Regensburg
Dr.	Susanne	Büsing	Zentrum für Kinder- und Jugendmedizin	Christliches Kinderhospital Osnabrück
Dr.	Doris	Dieninghoff	Lungenklinik Merheim	Kliniken der Stadt Köln
Dr.	Stefanie	Dillenhöfer	St. Josef Hospital - Klinik für Kinder- und Jugendmedizin - Christiane Herzog Zentrum	UKRUB - Katholisches Klinikum Bochum
Prof. Dr.	Anna-Maria	Dittrich	Klinik für Pädiatrische Pneumologie Christiane Herzog-Zentrum	Medizinische Hochschule Hannover (MHH)
Prof. Dr.	Helmut	Ellemunter	Tirol Kliniken GmbH Kinder- und Jugendheil- kunde - Mukoviszidose-Zentrum	Medizinische Universität Innsbruck
Dr.	Sebastian	Fähndrich	Klinik für Pneumologie	Universitätsklinikum Freiburg
Prof. Dr.	Rainald	Fischer		Lungenheilkunde München Pasing, München
Dr.	Norbert	Geier	Klinik für Kinder- und Jugendmedizin Klinikum am Gesundbrunnen, Perinatalzentrum	SLK-Kliniken Heilbronn
Dr.	Ute	Graepler-Mainka	Klinik für Kinder- und Jugendmedizin	Universitätsklinikum Tübingen
Prof. Dr.	Matthias	Griese	Kinderklinik und Kinderpoliklinik im Dr. von Haunerschen Kinderspital	LMU Klinikum der Universität München
Dr.	Jörg	Große-Onnebrink	Klinik für Kinder- und Jugendmedizin Allgemeine Pädiatrie	Universitätsklinikum Münster UKM
Dr.	Jutta	Hammermann	Haus 21 Universitäts-Mukoviszidose-Centrum (UMC)	Universitätsklinikum Carl Gustav Carusc, Dresden
Prof. Dr.	Helge	Hebestreit	Kinderpoliklinik Christiane Herzog-Zentrum Unterfranken	Universitätsklinikum Würzburg
Prof. Dr.	Andrea	Heinzmann	Zentrum für Kinder- und Jugendmedizin	Universitätsklinikum Freiburg
Dr.	Inka	Held	Praxis Kinderärzte im Friesenweg CF Centrum Altona	Kinder- und Jugendärztliche Gemeinschaftspraxis, Hamburg
Dr.	Manfred	Käding	Praxis für Kinder- und Jugendmedizin	Poliklinik Chemnitz
Dr.	Petra	Kaiser-Labusch	Klinik für Kinder- und Jugendmedizin Christiane Herzog-Zentrum	Eltern-Kind-Zentrum Prof. Hess, Bremen
Prof. Dr.	Wolfgang	Kamin	Klinik für Kinder- und Jugendmedizin Pulmologie/Allergologie	Evangelisches Krankenhaus Hamm (EVK)
Dr.	Axel	Kempa	Fachklinik Löwenstein	SLK-Kliniken Heilbronn, Löwenstein
Dr.	Birte	Kinder	Klinik für Kinder- und Jugendmedizin	Dietrich Bonhoeffer Klinikum, Neubrandenburg
Dr.	Cordula	Koerner-Rettberg	Klinik für Kinder- und Jugendmedizin	Marien Hospital Wesel gGmbH
Dr.	Holger	Köster	Klinik für Pädiatrische Pneumologie und Allergologie, Neonatologie und Intensivmedizin	Klinikum Oldenburg AöR
Dr.	Markus	Kratz	Klinik für Kinder- und Jugendmedizin Baden-Baden Balg	Klinkum Mittelbaden gGmbH, Baden-Baden
Dr.	Stefan	Kuhnert	Medizinische Klinik und Poliklinik II	Universitätsklinikum Gießen
Dr.	Peter	Küster	Kinder- und Jugendmedizin	Clemenshospital Münster
Dr.	Kerstin	Landwehr	Klinik für Kinder- und Jugendmedizin Schwerpunkt Kinder-Pneumologie	Evangelisches Klinikum Bethel, Bielefeld
Dr.	Simone	Lehmkühler	Klinik für Kinder- und Jugendmedizin Pädiatrische Pneumologie und Allergologie	Universitätsmedizin Mannheim
	Michael	Lorenz	Klinik für Kinder- und Jugendmedizin	Universitätsklinikum Jena
Dr.	Eva	Lücke	Klinik für Pneumologie	Otto-von-Guericke-Universität Magdeburg
Dr.	Rolf	Mahlberg	Innere Medizin 1	Klinikum Mutterhaus der Borromäerinnen, Trier
Prof. Dr.	Jochen	Mainz	Klinikum Westbrandenburg Pädiatrische Pulmologie und Allergologie	Medizinische Hochschule Brandenburg (MHB)

Dr.	Jochen	Meister	Klinik für Kinder- und Jugendmedizin	HELIOS Klinikum Aue
Dr.	Susanne	Nährig	Campus Innenstadt Medizinische Klinik - Pneumologie	LMU Klinikum der Universität München
Prof. Dr.	Lutz	Nährlich	Zentrum für Kinderheilkunde und Jugendmed. Abteilung Allgemeine Pädiatrie und Neonatologie	Universitätsklinikum Gießen-Marburg GmbH
Prof. Dr.	Tim	Niehues	Zentrum für Kinder- und Jugendmedizin	Helios Klinikum Krefeld
Dr.	Oliver	Nitsche	Klinik für Kinder- und Jugendmedizin Pädiatrische Pneumologie und Allergologie	Universitätsmedizin Mainz
Dr.	Anna	Nolde	II. Medizinische Klinik und Poliklinik Sektion Pneumologie	Universitätsklinikum Eppendorf UKE, Hamburg,
PD Dr.	Thomas	Nüßlein	Klinik für Kinder- und Jugendmedizin	Gemeinschaftsklinikum Mittelrhein, Koblenz
Dr.	Claus	Pfannenstiel		Kinderarztpraxis Laurensberg Aachen
	Anne	Pfülb	Klinik für Kinder- und Jugendmedizin	Klinikum Memmingen
Dr.	Margarethe	Pohl	Zentrum für Kinder- und Jugendgesundheit Kinderpneumologie	Kinderklinik Dritter Orden, Passau
Dr.	Krystyna	Poplawska	Fachkliniken Wangen Klinik für Pneumologie	Waldburg Zeil Kliniken, Wangen
PD Dr.	Freerk	Prenzel	Klinik und Poliklinik für Kinder- und Jugendmedizin	Universitätsklinikum Leipzig
Dr.	Katharina	Remke	Klinik für Allgemeine Pädiatrie und Neonatologie	Universitätsklinikum des Saarlandes, Homburg
PD Dr.	Felix C.	Ringshausen	Klinik für Pneumologie	Medizinische Hochschule Hannover (MHH)
Prof. Dr.	Markus A.	Rose	Zentrum für chronische Lungenerkrankungen Christiane Herzog Transitionszentrum	Klinikum Stuttgart – Olgahospital, Stuttgart
Prof. Dr.	Josef	Rosenecker	Fachkliniken Wangen Rehabilitationsklinik für Kinder und Jugendliche	Waldburg-Zeil Kliniken, Wamgen
Dr.	Friederike	Ruf	Pneumologie und Beatmungsmedizin	Robert Bosch Krankenhaus RBK, Stuttgart
Dr.	Anette	Scharschinger	Klinik für Kinder- und Jugendmedizin Kinderpneumologie und Allergologie	Universitätsklinikum Augsburg
PD Dr.	Sebastian	Schmidt	Klinik für Kinder- und Jugendmedizin	Universitätsmedizin Greifswald
PD Dr.	Sabina	Schmitt-Grohé	Kinderklinik - Sozialpädiatrisches Zentrum	Universitätsklinikum Erlangen
Dr.	Gudrun	Schopper	Klinik für Kinder- und Jugendmedizin Allergologie, Pneumologie, Umweltmedizin	München Klinik Schwabing, München
Dr.	Dirk	Schramm	Klinik für Kinder und Jugendliche Kinderpneumologie und Allergologie	Evangelisches Krankenhaus Düsseldorf
Prof. Dr.	Carsten	Schwarz	Kinder- und Jugendklinik	Klinikum Westbrandenburg gGmbH, Potsdam
Dr.	Christina	Smaczny	Klinik für Kinder- und Jugendmedizin, Christiane Herzog CF-Zentrum	Universitätsklinikum Frankfurt
Prof. Dr.	Olaf	Sommerburg	Sektion Pädiatrische Pneumologie und Allergologie	Universitätsklinikum Heidelberg
Dr.	Dana	Spittel	Kinder- und Jugendmedizin	Helios Klinikum Erfurt
Prof. Dr.	Mirjam	Stahl	Klinik für Pädiatrie mo S. Pneumologie und Immunologie - Christiane Herzog-Zentrum	Charité - Universitätsmedizin Berlin
Dr.	Dirk	Steffen	Innere Medizin	Luisenhospital Aachen
Prof. Dr.	Florian	Stehling	Klinik für Kinderheilkunde III Christiane Herzog Centrum Ruhr	Universitätsklinikum Essen (AöR)
Dr.	Simone	Stolz	Akademisches Lehrkrankenhaus der Charité Klinik für Kinder- und Jugendmedizin	Carl-Thiem-Klinikum Cottbus
Dr.	Sivagurunathan	Sutharsan	Ruhrlandklinik - Klinik für Pneumologie, Christiane Herzog-Zentrum	Universitätsmedizin Essen
Prof. Dr.	Tobias	Tenenbaum	Klinik für Kinder- und Jugendmedizin	Sana Klinkum Lichtenberg, Berlin
Dr.	Tina	Teßmer	Klinik für Kinder- und Jugendmedizin	Klinikum Worms gGmbH
PD Dr.	Wolfgang	Thomas	Kinder- und Jugendmedizin	Klinikum Mutterhaus der Borromäerinnen, Trier
Dr.	Christian	Timke	Kinderklinik und Jugendmedizin Christiane Herzog Zentrum Nord	Städtisches Krankenhaus Kiel
PD Dr.	Silke	van Koningsbruggen-Rietschel	Klinik für Kinder- und Jugendmedizin	Universitätsklinikum Köln
Dr.	Paul	Vöhringer	Klinik für Kinder- und Jugendmedizin Pneumologie	Städtisches Klinikum Karlsruhe

Dr.	Sabine	Wege	Thoraxklinik Heidelberg gGmbH Pneumologie und Beatmungstherapie	Universitätsklinik Heidelberg
Dr.	Britta	Welzenbach	Klinik für Kinder- und Jugendmedizin Sozialpädagogisches Zentrum (SPZ)	KJF Klinik Josefinum Augsburg
PD Dr.	Claudius	Werner	Kinder- und Jugendmedizin	Helios Kliniken Schwerin
Prof. Dr.	Heinrike	Wilkens	Innere Medizin V - Pneumologie, Allergologie Beatmungs- und Umweltmedizin	Universitätsklinikum des Saarlandes. Homburg
Dr.	Bettina	Wollschläger	Medizinische Fakultät der Klinik für Innere Medizin I	Universitätsklinikum Halle (Saale) UKH, Halle
Dr.	Sabine	Zirlik	Medizinische Klinik 1 Schwerpunkt Pneumologie	Universitätsklinikum Erlangen

FIGURE S1 Median change in percent predicted forced expiratory volume in 1 second ($ppFEV_1$) from the 6 months before to four 6-month periods after elexacaftor/tezacaftor/ivacaftor (ETI) initiation based on the type of mutation and by age group.

Changes (Δ) represent the median of individual differences between the 6-month (half-year) period preceding treatment and the corresponding 6-month periods after treatment initiation for participants with ppFEV1 values from the 6-month period before therapy initiation to the fourth 6-month period after starting treatment.

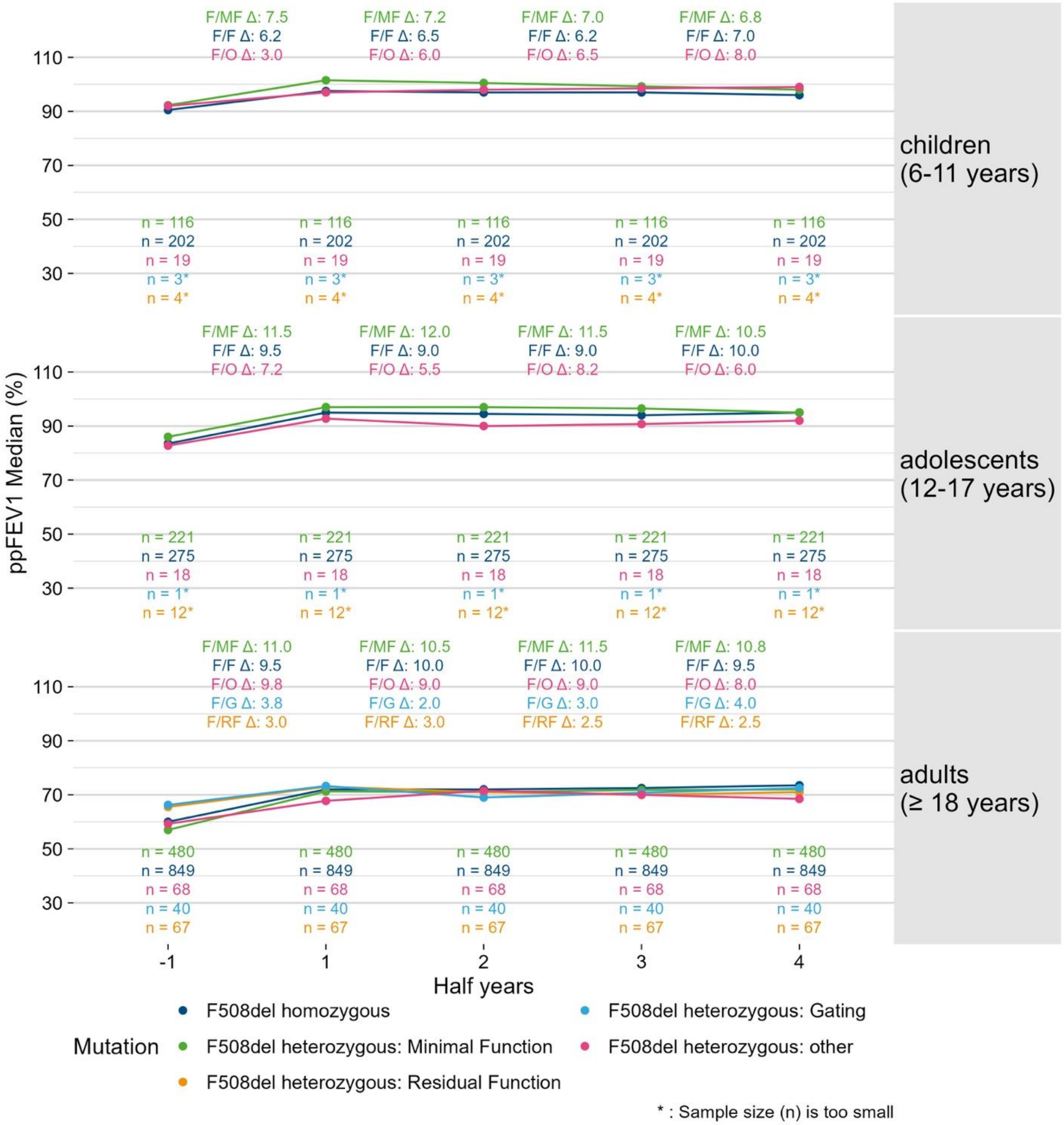

FIGURE S2 Median change in percent predicted forced expiratory volume in 1 second ($ppFEV_1$) from the 6 months before to four 6-month periods after elexacaftor/tezacaftor/ivacaftor (ETI) initiation based on previous modulator therapy and type of mutation.

Changes (Δ) represent the median of individual differences between the 6-month (half-year) period preceding treatment and the corresponding 6-month periods after treatment initiation for participants with $ppFEV_1$ values from the 6-month period before therapy initiation to the fourth 6-month period after starting treatment. Data are not shown if subgroup sample size was <15.

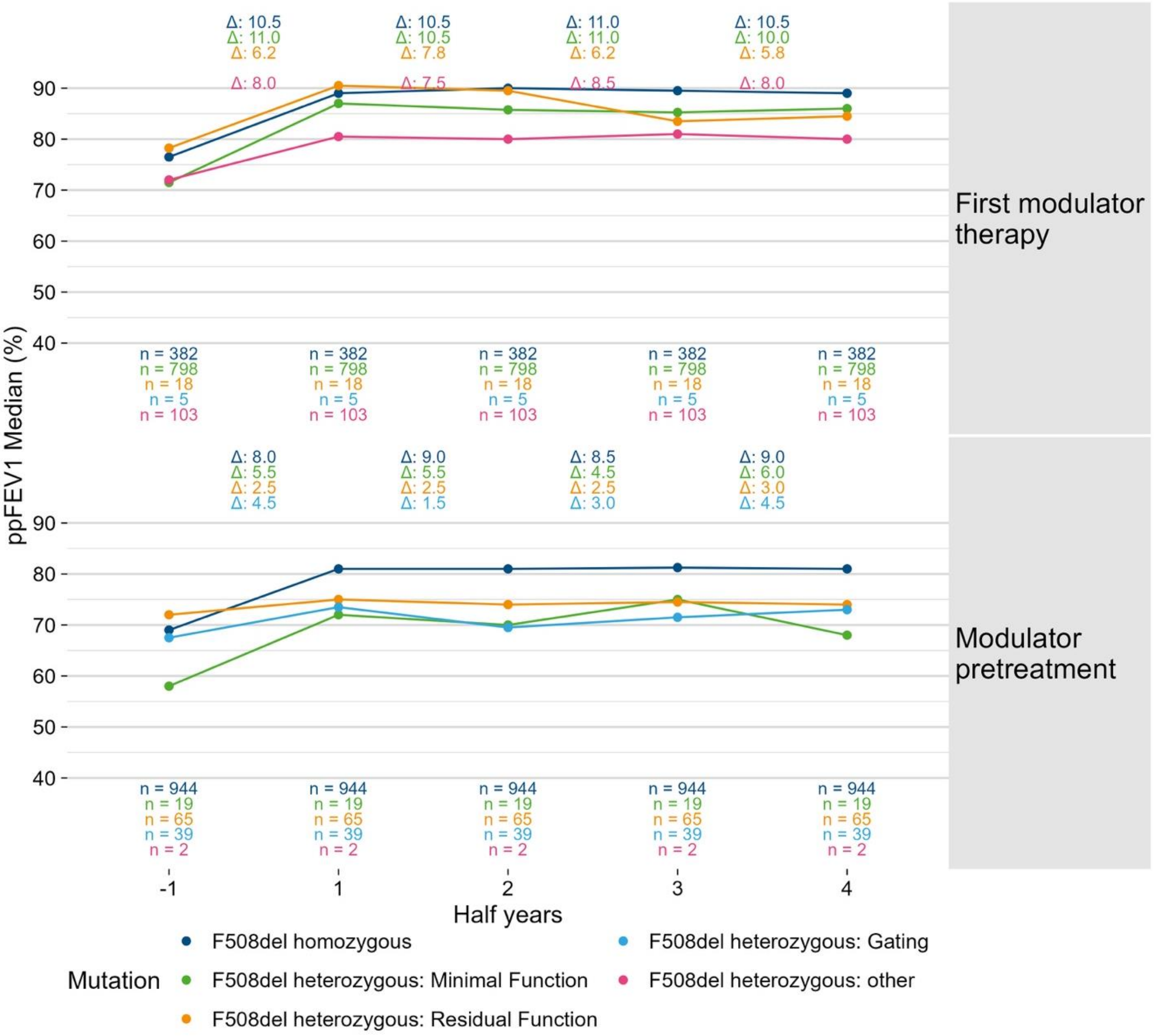

FIGURE S3 Median change in percent predicted forced mid-expiratory flow (FEF25-75) from the 6 months before to four 6-month periods after elexacaftor/tezacaftor/ivacaftor (ETI) initiation in subgroups based on baseline percent predicted forced expiratory volume in 1 second ($ppFEV_1$) and by age group.

Changes (Δ) represent the median of individual differences between the 6-month (half-year) period preceding treatment and the respective 6-month period after treatment initiation.

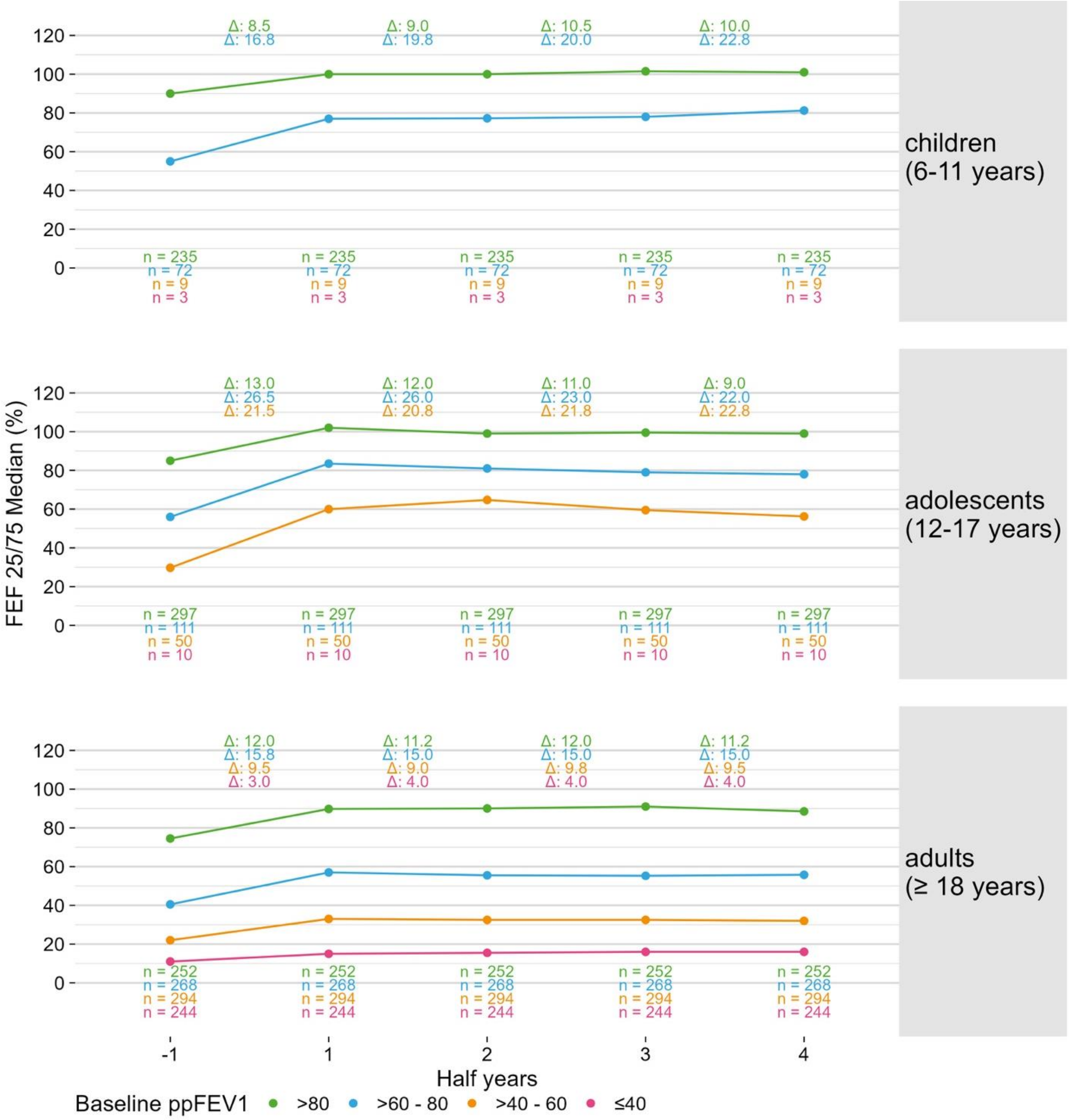

TABLE S1.1 Change in percent predicted forced expiratory volume in 1 second in children by subgroup

Time period	Variable	Children (age 6–11 years)			
		ppFEV$_1$			
		Median (IQR)	Δ (IQR)	N	p-value
0–6 mo before ETI		92.0 (80.0;100.6)		344	
0–6 mo on ETI		98.0 (88.4;108.0)	6.5 (0.4;13.1)	344	<0.001
7–12 mo on ETI		98.0 (90.0;106.5)	6.5 (;0.5;13.0)	344	<0.001
13–18 mo on ETI		98.0 (89.0;107.0)	6.5 (0.0;14.1)	344	<0.001
18–24 mo on ETI		97.5 (88.0;107.2)	6.5 (;0.6;14.0)	344	<0.001
**CFTR modulator pretreatment**					
0–6 mo before ETI	naïve	91.5 (79.0;101.0)		179	
0–6 mo on ETI	naïve	98.5 (88.5;109.0)	7.0 (0.0;14.0)	179	<0.001
7–12 mo on ETI	naïve	98.5 (90.0;106.5)	6.5 (;1.5;14.0)	179	<0.001
13–18 mo on ETI	naïve	98.0 (88.8;107.0)	6.5 (0.0;15.2)	179	<0.001
18–24 mo on ETI	naïve	98.0 (88.5;108.0)	6.0 (;0.2;15.0)	179	<0.001
0–6 mo before ETI	pretreated	92.0 (80.5;100.0)		165	
0–6 mo on ETI	pretreated	97.5 (88.5;106.5)	6.0 (0.5;12.0)	165	<0.001
7–12 mo on ETI	pretreated	97.0 (89.5;105.5)	6.5 (1.0;12.0)	165	<0.001
13–18 mo on ETI	pretreated	97.0 (89.0;106.0)	6.5 (0.5;13.5)	165	<0.001
18–24 mo on ETI	pretreated	96.0 (87.0;106.0)	7.0 (;1.0;12.5)	165	<0.001
**Baseline ppFEV$_1$**					
0–6 mo before ETI	≤40	37.0 (35.0;37.2)		3	
0–6 mo on ETI	≤40	65.0 (54.0;74.0)	27.5 (16.8;38.8)	3	
7–12 mo on ETI	≤40	85.0 (67.0;94.5)	47.5 (29.8;59.2)	3	
13–18 mo on ETI	≤40	71.0 (57.5;82.5)	33.5 (20.2;47.2)	3	
18–24 mo on ETI	≤40	75.5 (60.0;84.2)	38.0 (22.8;49.0)	3	
0–6 mo before ETI	>40–60	53.0 (49.9;58.2)		10	
0–6 mo on ETI	>40–60	80.8 (71.6;84.6)	21.5 (18.6;28.8)	10	
7–12 mo on ETI	>40–60	83.5 (79.9;94.0)	27.5 (23.6;37.2)	10	
13–18 mo on ETI	>40–60	81.5 (70.1;90.8)	27.2 (20.0;31.5)	10	
18–24 mo on ETI	>40–60	86.0 (71.4;94.4)	26.8 (20.6;38.5)	10	
0–6 mo before ETI	>60–80	73.5 (66.9;77.0)		76	
0–6 mo on ETI	>60–80	86.2 (78.0;96.1)	13.0 (7.4;24.6)	76	<0.001
7–12 mo on ETI	>60–80	87.8 (77.0;93.5)	14.5 (6.9;23.2)	76	<0.001
13–18 mo on ETI	>60–80	85.5 (78.9;94.0)	14.2 (6.9;22.1)	76	<0.001
18–24 mo on ETI	>60–80	85.2 (77.4;92.6)	14.0 (6.4;22.2)	76	<0.001
0–6 mo before ETI	>80	97.0 (89.0;103.2)		255	
0–6 mo on ETI	>80	102.0 (93.0;109.5)	4.5 (–0.2;10.0)	255	<0.001
7–12 mo on ETI	>80	100.5 (94.2;109.0)	4.5 (–2.0;10.0)	255	<0.001
13–18 mo on ETI	>80	101.0 (93.8;110.0)	4.0 (–1.0;11.0)	255	<0.001
18–24 mo on ETI	>80	101.5 (93.0;109.5)	4.5 (–2.0;10.5)	255	<0.001
**Mutation**					
0–6 mo before ETI	F/F	90.5 (79.6;101.0)		202	
0–6 mo on ETI	F/F	97.5 (87.0;106.9)	6.2 (0.0;12.9)	202	<0.001
7–12 mo on ETI	F/F	97.0 (87.8;107.0)	6.5 (0.5;12.4)	202	<0.001
13–18 mo on ETI	F/F	97.0 (88.1;106.8)	6.2 (0.6;13.5)	202	<0.001
18–24 mo on ETI	F/F	96.0 (87.2;106.0)	7.0 (–1.0;13.0)	202	<0.001
0–6 mo before ETI	F/MF	92.2 (79.0;101.2)		116	

0–6 mo on ETI	F/MF	101.5 (91.0;109.1)	7.5 (1.5;15.0)	116	<0.001
7–12 mo on ETI	F/MF	100.5 (91.0;106.5)	7.2 (–0.5;13.2)	116	<0.001
13–18 mo on ETI	F/MF	99.2 (89.4;107.1)	7.0 (–1.0;15.8)	116	<0.001
18–24 mo on ETI	F/MF	98.0 (89.9;108.1)	6.8 (0.0;16.5)	116	<0.001
0–6 mo before ETI	F/RF	95.5 (91.1;103.4)		4	
0–6 mo on ETI	F/RF	96.5 (94.8;101.9)	1.0 (–1.5;3.6)	4	
7–12 mo on ETI	F/RF	95.0 (93.2;101.8)	1.2 (0.1;2.1)	4	
13–18 mo on ETI	F/RF	98.5 (93.8;105.8)	2.2 (1.5;2.8)	4	
18–24 mo on ETI	F/RF	96.5 (93.5;103.8)	1.2 (–1.1;4.1)	4	
0–6 mo before ETI	F/G	96.5 (87.8;98.2)		3	
0–6 mo on ETI	F/G	98.0 (93.0;100.0)	5.5 (1.8;7.2)	3	
7–12 mo on ETI	F/G	98.0 (95.8;98.0)	1.5 (–0.2;8.0)	3	
13–18 mo on ETI	F/G	94.0 (89.2;95.2)	–2.5 (–3.0;1.5)	3	
18–24 mo on ETI	F/G	92.0 (87.5;95.5)	–1.0 (–2.8;1.5)	3	
0–6 mo before ETI	F/O	92.0 (83.5;97.0)		19	
0–6 mo on ETI	F/O	97.0 (85.2;105.2)	3.0 (–0.2;9.5)	19	
7–12 mo on ETI	F/O	98.0 (83.5;106.0)	6.0 (–3.8;14.5)	19	
13–18 mo on ETI	F/O	98.5 (91.2;106.2)	6.5 (0.8;18.5)	19	
18–24 mo on ETI	F/O	99.0 (87.0;106.5)	8.0 (2.5;15.0)	19	

Δ, change; CFTR, cystic fibrosis transmembrane conductance regulator; ETI, elexacaftor-tezacaftor-ivacftor; F/F, F508del homozygous; F/MF, F508del heterozygous: minimal function; F/RF, F508del heterozygous: residual function; F/G, F508del heterozygous: gating; F/O, F508del heterozygous: other; IQR, interquartile range; mo, months; ppFEV$_1$, percent predicted forced expiratory volume in 1 second.

TABLE S1.2 Change in percent predicted forced expiratory volume in 1 second in adolescents by subgroup

**Time period**	**Variable**	**Adolescents (age 12–17 years)**			
		**ppFEV$_1$**			
		**Median (IQR)**	**Δ (IQR)**	**N**	**p-value**
0–6 mo before ETI		85.0 (73.0;95.0)		527	
0–6 mo on ETI		96.0 (85.5;105.5)	10.0 (3.5;18.0)	527	<0.001
7–12 mo on ETI		96.0 (85.5;105.0)	10.0 (3.0;18.0)	527	<0.001
13–18 mo on ETI		95.5 (84.5;105.0)	10.0 (3.0;17.8)	527	<0.001
18–24 mo on ETI		95.0 (84.0;104.0)	10.0 (3.0;18.0)	527	<0.001
**CFTR modulator pretreatment**					
0–6 mo before ETI	naïve	86.0 (73.0;95.5)		340	
0–6 mo on ETI	naïve	97.0 (87.5;106.5)	11.0 (5.0;19.5)	340	<0.001
7–12 mo on ETI	naïve	97.5 (87.5;106.0)	11.2 (3.5;19.1)	340	<0.001
13–18 mo on ETI	naïve	97.0 (87.4;106.0)	11.0 (4.0;19.1)	340	<0.001
18–24 mo on ETI	naïve	95.8 (86.4;107.0)	10.5 (3.4;20.0)	340	<0.001
0–6 mo before ETI	pretreated	83.0 (73.0;92.8)		187	
0–6 mo on ETI	pretreated	94.0 (81.5;104.0)	8.5 (2.5;15.8)	187	<0.001
7–12 mo on ETI	pretreated	93.0 (81.8;104.5)	8.5 (3.0;15.0)	187	<0.001
13–18 mo on ETI	pretreated	91.5 (82.0;102.8)	7.0 (2.2;14.8)	187	<0.001
18–24 mo on ETI	pretreated	93.0 (83.0;102.0)	8.0 (2.2;14.0)	187	<0.001
**Baseline ppFEV$_1$**					
0–6 mo before ETI	≤40	32.2 (29.8;36.1)		12	
0–6 mo on ETI	≤40	55.0 (46.9;62.8)	21.8 (16.4;32.1)	12	

7–12 mo on ETI	≤40	56.5 (44.8;60.0)	21.5 (13.0;28.1)	12	
13–18 mo on ETI	≤40	55.0 (48.0;61.6)	24.2 (14.8;26.9)	12	
18–24 mo on ETI	≤40	56.5 (46.0;67.2)	28.0 (13.1;30.8)	12	
0–6 mo before ETI	>40–60	54.5 (48.4;58.0)		56	
0–6 mo on ETI	>40–60	71.0 (66.0;82.4)	16.8 (11.1;30.0)	56	<0.001
7–12 mo on ETI	>40–60	73.0 (66.0;87.5)	19.0 (10.4;31.9)	56	<0.001
13–18 mo on ETI	>40–60	72.5 (64.0;86.0)	19.0 (11.2;32.5)	56	<0.001
18–24 mo on ETI	>40–60	71.0 (63.6;83.6)	20.2 (10.4;29.9)	56	<0.001
0–6 mo before ETI	>60–80	73.0 (68.0;77.2)		131	
0–6 mo on ETI	>60–80	88.0 (80.8;95.0)	15.5 (10.0;23.0)	131	<0.001
7–12 mo on ETI	>60–80	87.0 (80.5;96.0)	15.0 (8.0;25.0)	131	<0.001
13–18 mo on ETI	>60–80	88.5 (80.5;97.2)	14.5 (9.2;24.0)	131	<0.001
18–24 mo on ETI	>60–80	87.0 (81.0;95.0)	15.0 (8.8;22.5)	131	<0.001
0–6 mo before ETI	>80	92.0 (86.9;100.1)		328	
0–6 mo on ETI	>80	102.0 (94.5;109.1)	6.5 (2.0;13.0)	328	<0.001
7–12 mo on ETI	>80	101.5 (94.0;109.0)	6.8 (2.0;14.0)	328	<0.001
13–18 mo on ETI	>80	100.2 (92.0;109.0)	6.0 (1.0;12.6)	328	<0.001
18–24 mo on ETI	>80	101.0 (92.9;109.0)	6.0 (0.0;13.0)	328	<0.001
**Mutation**					
0–6 mo before ETI	F/F	83.5 (73.2;94.0)		275	
0–6 mo on ETI	F/F	95.0 (83.8;104.5)	9.5 (3.5;16.8)	275	<0.001
7–12 mo on ETI	F/F	94.5 (85.0;104.8)	9.0 (3.5;16.5)	275	<0.001
13–18 mo on ETI	F/F	94.0 (84.0;104.0)	9.0 (3.5;17.2)	275	<0.001
18–24 mo on ETI	F/F	95.0 (84.0;103.8)	10.0 (3.0;16.2)	275	<0.001
0–6 mo before ETI	F/MF	86.0 (70.0;96.0)		221	
0–6 mo on ETI	F/MF	97.0 (87.0;106.5)	11.5 (4.0;20.0)	221	<0.001
7–12 mo on ETI	F/MF	97.0 (87.5;106.0)	12.0 (3.5;20.0)	221	<0.001
13–18 mo on ETI	F/MF	96.5 (86.0;106.0)	11.5 (3.0;19.5)	221	<0.001
18–24 mo on ETI	F/MF	95.0 (84.5;106.0)	10.5 (2.0;20.5)	221	<0.001
0–6 mo before ETI	F/RF	95.8 (87.4;110.2)		12	
0–6 mo on ETI	F/RF	102.2 (96.8;116.4)	7.0 (4.9;11.6)	12	
7–12 mo on ETI	F/RF	107.2 (95.0;114.0)	8.0 (2.5;9.9)	12	
13–18 mo on ETI	F/RF	105.5 (91.0;116.9)	5.8 (2.2;10.6)	12	
18–24 mo on ETI	F/RF	101.5 (94.8;113.2)	6.0 (1.8;11.0)	12	
0–6 mo before ETI	F/G	88.0 (88.0;88.0)		1	
0–6 mo on ETI	F/G	99.5 (99.5;99.5)	11.5	1	
7–12 mo on ETI	F/G	71.0 (71.0;71.0)	–17.	1	
13–18 mo on ETI	F/G	97.0 (97.0;97.0)	9.0	1	
18–24 mo on ETI	F/G	101.0 (101.0;101.0)	13.0	1	
0–6 mo before ETI	F/O	82.8 (77.4;91.9)		18	
0–6 mo on ETI	F/O	92.8 (80.9;102.5)	7.2 (–4.5;13.1)	18	
7–12 mo on ETI	F/O	90.0 (82.0;99.1)	5.5 (–0.6;9.2)	18	
13–18 mo on ETI	F/O	90.8 (80.5;100.0)	8.2 (–1.5;10.1)	18	
18–24 mo on ETI	F/O	92.0 (80.6;94.5)	6.0 (–2.5;12.6)	18	

Δ, change; CFTR, cystic fibrosis transmembrane conductance regulator; ETI, elexacaftor-tezacaftor-ivacftor; F/F, F508del homozygous; F/MF, F508del heterozygous: minimal function; F/RF, F508del heterozygous: residual function; F/G, F508del heterozygous: gating; F/O, F508del heterozygous: other; IQR, interquartile range; mo, months; $ppFEV_1$, percent predicted forced expiratory volume in 1 second.

TABLE S1.3 Change in percent predicted forced expiratory volume in 1 second in adults by subgroup

Time period	Variable	Adults (age ≥18 years)			
		ppFEV$_1$			
		Median (IQR)	Δ (IQR)	N	p-value
0–6 mo before ETI		59.5 (41.4;77.5)		1504	
0–6 mo on ETI		72.0 (52.0;89.5)	9.5 (4.5;16.5)	1504	<0.001
7–12 mo on ETI		71.5 (53.0;89.0)	10.0 (4.0;16.5)	1504	<0.001
13–18 mo on ETI		72.0 (52.5;89.0)	9.8 (3.5;16.5)	1504	<0.001
18–24 mo on ETI		72.5 (52.5;89.0)	9.0 (4.0;16.0)	1504	<0.001
**CFTR modulator pretreatment**					
0–6 mo before ETI	naïve	61.0 (42.0;79.0)		787	
0–6 mo on ETI	naïve	75.0 (55.0;92.0)	10.5 (5.5;18.0)	787	<0.001
7–12 mo on ETI	naïve	75.0 (54.5;92.0)	10.5 (5.0;18.0)	787	<0.001
13–18 mo on ETI	naïve	76.0 (55.2;92.0)	11.0 (5.0;18.0)	787	<0.001
18–24 mo on ETI	naïve	75.0 (55.0;91.5)	10.5 (4.5;18.0)	787	<0.001
0–6 mo before ETI	pretreated	58.0 (41.0;75.0)		717	
0–6 mo on ETI	pretreated	69.5 (49.5;87.0)	8.0 (3.0;14.5)	717	<0.001
7–12 mo on ETI	pretreated	69.0 (51.0;86.0)	8.5 (3.0, 14.5)	717	<0.001
13–18 mo on ETI	pretreated	69.0 (51.0;86.0)	8.0 (2.5;15.0)	717	<0.001
18–24 mo on ETI	pretreated	68.0 (51.0;85.0)	8.0 (3.0;14.0)	717	<0.001
**Baseline ppFEV$_1$**					
0–6 mo before ETI	≤40	32.2 (28.0;37.0)		356	
0–6 mo on ETI	≤40	41.0 (34.0;48.0)	8.2 (5.0;15.0)	356	<0.001
7–12 mo on ETI	≤40	41.8 (35.5;49.5)	10.0 (5.5;16.0)	356	<0.001
13–18 mo on ETI	≤40	41.5 (35.0;49.1)	10.0 (4.5;16.0)	356	<0.001
18–24 mo on ETI	≤40	41.2 (34.0;49.0)	9.8 (5.0;16.0)	356	<0.001
0–6 mo before ETI	>40–60	50.0 (45.5;55.0)		416	
0–6 mo on ETI	>40–60	62.5 (54.0;70.6)	11.5 (5.5;19.5)	416	<0.001
7–12 mo on ETI	>40–60	62.5 (55.0;71.0)	11.2 (5.5;19.5)	416	<0.001
13–18 mo on ETI	>40–60	62.2 (54.0;71.0)	11.8 (5.0;20.0)	416	<0.001
18–24 mo on ETI	>40–60	62.5 (54.4;71.0)	11.5 (5.0;19.5)	416	<0.001
0–6 mo before ETI	>60–80	69.5 (65.0;74.0)		400	
0–6 mo on ETI	>60–80	81.8 (74.0;88.5)	10.8 (5.5;18.6)	400	<0.001
7–12 mo on ETI	>60–80	81.5 (73.4;88.1)	11.0 (4.5;17.0)	400	<0.001
13–18 mo on ETI	>60–80	81.5 (73.5;88.1)	11.0 (5.0;17.5)	400	<0.001
18–24 mo on ETI	>60–80	81.0 (74.0;88.0)	10.5 (4.5;17.1)	400	<0.001
0–6 mo before ETI	>80	92.0 (85.9;100.0)		332	
0–6 mo on ETI	>80	100.8 (92.5;109.0)	6.0 (1.0;12.5)	332	<0.001
7–12 mo on ETI	>80	100.5 (92.0;108.0)	6.0 (0.5;12.0)	332	<0.001
13–18 mo on ETI	>80	100.0 (91.9;108.1)	6.0 (1.0;11.5)	332	<0.001
18–24 mo on ETI	>80	99.0 (91.5;107.6)	5.5 (0.5;11.0)	332	<0.001

**Mutation**					
0–6 mo before ETI	F/F	60.0 (42.0;77.5)		849	
0–6 mo on ETI	F/F	72.0 (52.0;90.0)	9.5 (4.5;16.5)	849	<0.001
7–12 mo on ETI	F/F	72.0 (53.5;89.5)	10.0 (4.5;17.0)	849	<0.001
13–18 mo on ETI	F/F	72.5 (53.0;89.5)	10.0 (4.5;16.5)	849	<0.001
18–24 mo on ETI	F/F	73.5 (53.0;89.0)	9.5 (4.5;16.0)	849	<0.001
0–6 mo before ETI	F/MF	57.0 (39.4;75.6)		480	
0–6 mo on ETI	F/MF	71.2 (52.0;90.0)	11.0 (5.5;18.1)	480	<0.001
7–12 mo on ETI	F/MF	71.0 (52.9;89.2)	10.5 (5.0;18.5)	480	<0.001
13–18 mo on ETI	F/MF	71.8 (52.4;90.0)	11.5 (4.5;18.5)	480	<0.001
18–24 mo on ETI	F/MF	72.0 (51.9;90.0)	10.8 (4.5;18.5)	480	<0.001
0–6 mo before ETI	F/RF	65.5 (44.2;82.2)		67	
0–6 mo on ETI	F/RF	73.0 (47.8;86.5)	3.0 (–0.5;7.0)	67	0.002
7–12 mo on ETI	F/RF	71.0 (47.2;88.2)	3.0 (0.2;6.5)	67	0.001
13–18 mo on ETI	F/RF	70.0 (47.2;86.0)	2.5 (0.2;6.0)	67	0.001
18–24 mo on ETI	F/RF	71.0 (49.2;87.0)	2.5 (–1.2;7.0)	67	0.021
0–6 mo before ETI	F/G	66.2 (47.1;83.1)		40	
0–6 mo on ETI	F/G	73.2 (53.0;86.0)	3.8 (0.9;6.8)	40	0.001
7–12 mo on ETI	F/G	69.0 (53.8;84.1)	2.0 (–0.6;5.2)	40	0.110
13–18 mo on ETI	F/G	70.8 (53.8;84.4)	3.0 (–0.1;6.0)	40	0.007
18–24 mo on ETI	F/G	72.5 (53.9;83.6)	4.0 (0.0;6.8)	40	0.016
0–6 mo before ETI	F/O	59.2 (40.8;74.2)		68	
0–6 mo on ETI	F/O	67.8 (56.5;84.1)	9.8 (4.1;13.6)	68	<0.001
7–12 mo on ETI	F/O	71.5 (53.0;85.0)	9.0 (4.9;14.2)	68	<0.001
13–18 mo on ETI	F/O	70.0 (53.4;83.2)	9.0 (5.0;14.5)	68	<0.001
18–24 mo on ETI	F/O	68.5 (53.0;83.0)	8.0 (3.8;14.1)	68	<0.001

Δ, change; CFTR, cystic fibrosis transmembrane conductance regulator; ETI, elexacaftor-tezacaftor-ivacftor; F/F, F508del homozygous; F/MF, F508del heterozygous: minimal function; F/RF, F508del heterozygous: residual function; F/G, F508del heterozygous: gating; F/O, F508del heterozygous: other; IQR, interquartile range; mo, months; $ppFEV_1$, percent predicted forced expiratory volume in 1 second.

TABLE SX P-values of differences in Delta percent predicted forced expiratory volume in 1 second by subgroup

**Time period**	**Children vs. adolescents p-value**	**Children vs. adults p-value**	**Adolescents vs. adults p-value**	**Naïve vs. pretreatment p-value**
0–6 mo before ETI				
0–6 mo on ETI	<0.001	<0.001	>0.9	<0.001
7–12 mo on ETI	<0.001	<0.001	>0.9	<0.001
13–18 mo on ETI	0.002	<0.001	>0.9	<0.001
18–24 mo on ETI	0.001	<0.001	>0.9	<0.001

		**Baseline $ppFEV_1$**		
**Time Period**		**vs. >40-60**	**vs. >60–80**	**vs. >80**
0–6 mo before ETI	≤40			
0–6 mo on ETI	≤40	<0.001	0.001	<0.001

7–12 mo on ETI	≤40	0.087	>0.9	<0.001
13–18 mo on ETI	≤40	0.032	0.288	<0.001
18–24 mo on ETI	≤40	0.130	>0.9	<0.001
0–6 mo before ETI	>40–60			
0–6 mo on ETI	>40–60		>0.9	<0.001
7–12 mo on ETI	>40–60		>0.9	<0.001
13–18 mo on ETI	>40–60		>0.9	<0.001
18–24 mo on ETI	>40–60		>0.9	<0.001
0–6 mo before ETI	>60–80			
0–6 mo on ETI	>60–80			<0.001
7–12 mo on ETI	>60–80			<0.001
13–18 mo on ETI	>60–80			<0.001
18–24 mo on ETI	>60–80			<0.001

Mutation

**Time Period**		**vs. F/MF**	**vs. F/RF**	**vs. F/G**	**vs. F/O**
0–6 mo before ETI	F/F				
0–6 mo on ETI	F/F	0.006	<0.001	<0.001	>0.9
7–12 mo on ETI	F/F	0.299	<0.001	<0.001	>0.9
13–18 mo on ETI	F/F	0.110	<0.001	<0.001	>0.9
18–24 mo on ETI	F/F	0.299	<0.001	<0.001	>0.9
0–6 mo before ETI	F/MF				
0–6 mo on ETI	F/MF		<0.001	<0.001	0.060
7–12 mo on ETI	F/MF		<0.001	<0.001	0.225
13–18 mo on ETI	F/MF		<0.001	<0.001	0.532
18–24 mo on ETI	F/MF		<0.001	<0.001	0.420
0–6 mo before ETI	F/RF				
0–6 mo on ETI	F/RF			>0.9	0.025
7–12 mo on ETI	F/RF			>0.9	0.016
13–18 mo on ETI	F/RF			>0.9	<0.001
18–24 mo on ETI	F/RF			>0.9	0.009
0–6 mo before ETI	F/G				
0–6 mo on ETI	F/G				0.315
7–12 mo on ETI	F/G				0.007
13–18 mo on ETI	F/G				0.003
18–24 mo on ETI	F/G				0.032

Δ, change; CFTR, cystic fibrosis transmembrane conductance regulator; ETI, elexacaftor-tezacaftor-ivacftor; F/F, F508del homozygous; F/MF, F508del heterozygous: minimal function; F/RF, F508del heterozygous: residual function; F/G, F508del heterozygous: gating; F/O, F508del heterozygous: other; IQR, interquartile range; mo, months; ppFEV$_1$, percent predicted forced expiratory volume in 1 second.

TABLE S2 Participant demographic and clinical characteristics at baseline based on the change from baseline in percent predicted forced expiratory volume in 1 second during 2 years of elexacaftor-tezacaftor-ivacftor therapy in the overall population

	Change in $ppFEV_1$ from baseline		p-value[1]
	<5%	≥5%	
**Age, years**			0.080
N	542	1,833	
Median (IQR)	21.3 (12.1, 34.3)	23.3 (15.1, 33.1)	
Range	6.0, 74.0	6.0, 74.9	
**Sex, n (%)**			0.11
Male	300 (55.4)	904 (49.3)	
Female	242 (44.6)	929 (50.7)	
**$ppFEV_1$ at baseline, %**			<0.001
N	542	1,833	
Median (IQR)	86.0 (59.0, 99.0)	68.5 (49.0, 86.0)	
Range	13.0, 141.0	13.5, 124.0	
**$ppFEV_1$ during ETI therapy, %**			0.3
N	542	1,833	
Median (IQR)	84.5 (59.1, 97.4)	84.0 (64.0, 99.0)	
Range	17.0, 132.0	24.0, 135.0	
**ppFEF25-75 at baseline, %**			<0.001
N	478	1,604	
Median (IQR)	71.0 (34.0, 92.0)	44.8 (21.0, 73.5)	
Range	3.0, 172.0	3.0, 152.5	
**ppFEF25/75 during ETI therapy, %**			>0.9
N	506	1,607	
Median (IQR)	68.3 (31.1, 92.0)	66.0 (34.3, 95.8)	
Range	3.0, 175.0	6.0, 173.0	
**BMI at baseline, kg/m^2**			0.2
N	303	1,199	
Median (IQR)	21.5 (19.5, 23.7)	21.1 (19.2, 23.0)	
Range	15.0, 33.7	13.5, 36.3	
**BMI on ETI therapy, kg/m^2**			0.6
N	304	1,200	
Median (IQR)	22.1 (20.3, 24.4)	22.6 (20.6, 24.6)	
Range	15.0, 35.0	14.8, 38.7	
**BMI z-score at baseline**			0.2
N	238	633	
Median (IQR)	–0.3 (–0.9, 0.2)	–0.5 (–1.0, 0.2)	
Range	–2.6, 2.0	–5.5, 2.0	
**BMI z-score on ETI therapy**			>0.9
N	238	633	
Median (IQR)	–0.2 (–0.9, 0.5)	–0.2 (–0.8, 0.5)	
Range	–3.2, 2.3	–4.3, 2.2	
**Mutation, n (%)**			<0.001
F508del homozygous	276 (50.9)	1,050 (57.3)	
F508del heterozygous: minimal function	173 (31.9)	644 (35.1)	

	Change in ppFEV$_1$ from baseline		p-value[1]
	**<5%**	**≥5%**	
F508del heterozygous: residual function	44 (8.1)	39 (2.1)	
F508del heterozygous: gating	25 (4.6)	19 (1.0)	
F508del heterozygous: other	24 (4.4)	81 (4.4)	
**Exacerbation at baseline, n (%)**			<0.001
Yes	131 (24.2)	769 (42.0)	
No	411 (75.8)	1,064 (58.0)	
Unknown	0 (0.0)	0 (0.0)	
**Exacerbation on ETI therapy, n (%)**			>0.9
Yes	123 (22.7)	421 (23.0)	
No	419 (77.3)	1,412 (77.0)	
Unknown	0 (0.0)	0 (0.0)	
***Pseudomonas aeruginosa* at baseline, n (%)**			<0.001
Yes	196 (36.2)	888 (48.4)	
No	340 (62.7)	917 (50.0)	
Unknown	6 (1.1)	28 (1.5)	
***Pseudomonas aeruginosa* during ETI therapy, n (%)**			0.11
Yes	133 (24.5)	530 (28.9)	
No	409 (75.5)	1,290 (70.4)	
Unknown	0 (0.0)	13 (0.7)	
**CFTR modulator pretreatment, n (%)**	271 (50.0%)	798 (43.5%)	0.080

[1]Adjusted p-value using the Holm correction for multiple testing.

BMI, body mass index; CFTR, cystic fibrosis transmembrane conductance regulator; ETI, elexacaftor-tezacaftor-ivacftor; IQR, interquartile range; ppFEF25-75, percent predicted mid-expiratory flow; ppFEV1, percent predicted forced expiratory volume in 1 second.

TABLE S3 Participant demographic and clinical characteristics at baseline based on the change from baseline in percent predicted forced expiratory volume in 1 second during 2 years of elexacaftor-tezacaftor-ivacftor therapy within age groups.

	Children (age 6–11 years)			Adolescents (age 12–17 years)			Adults (age ≥18 years)		
	Change in $ppFEV_1$ from baseline			Change in $ppFEV_1$ from baseline			Change in $ppFEV_1$ from baseline		
	<5%	≥5%	p-value[1]	<5%	≥5%	p-value[1]	<5%	≥5%	p-value[1]
**Age, years**			>0.9			0.2			0.005
N	120	224		118	409		304	1,200	
Median (IQR)	9.1 (7.5, 10.5)	9.2 (7.5, 10.6)		13.7 (12.6, 15.8)	14.6 (13.0, 16.0)		32.7 (25.1, 41.4)	30.1 (23.6, 37.5)	
Range	6.0, 12.0	6.0, 12.0		12.0, 17.9	12.0, 18.0		18.2, 74.0	18.0, 74.9	
**Sex, n (%)**			>0.9			0.12			>0.9
Male	67 (55.%)	108 (48.2)		66 (55.9)	176 (43.0)		167 (54.9)	620 (51.7)	
Female	53 (44.2)	116 (51.8)		52 (44.1)	233 (57.0)		137 (45.1)	580 (48.3)	
**$ppFEV_1$ at baseline, %**			<0.001			<0.001			<0.001
N	120	224		118	409		304	1,200	
Median (IQR)	97.8 (89.0, 105.3)	89.0 (78.0, 97.5)		93.8 (84.5, 101.0)	84.0 (71.0, 93.0)		65.0 (42.4, 88.0)	58.0 (41.9, 74.5)	
Range	49.0, 141.0	37.0, 124.0		38.0, 131.0	27.0, 124.0		13.0, 121.5	13.5, 124.0	
**$ppFEV_1$ during ETI therapy, %**			0.007			0.018			0.001
N	120	224		118	409		304	1,200	
Median (IQR)	93.3 (85.9, 103.1)	100.0 (92.0, 108.6)		91.0 (84.1, 101.4)	96.5 (86.0, 106.0)		64.3 (44.8, 88.0)	74.0 (54.9, 89.1)	
Range	54.0, 132.0	44.0, 135.0		37.0, 131.0	35.0, 131.0		17.0, 121.5	24.0, 128.0	
**ppFEF25-75 at baseline, %**			<0.001			<0.001			0.003
N	116	213		114	385		248	1,006	
Median (IQR)	88.3 (73.5, 104.1)	78.0 (58.0, 94.0)		83.8 (71.0, 99.5)	72.0 (52.0, 88.0)		36.0 (18.0, 70.6)	29.0 (16.0, 48.0)	
Range	16.5, 172.0	13.0, 152.5		19.0, 140.0	6.0, 146.0		3.0, 145.0	3.0, 131.5	
**ppFEF25/75 during ETI therapy, %**			0.003			0.004			0.080
N	116	212		115	375		275	1,020	
Median (IQR)	87.3 (71.8, 103.6)	101.0 (80.5, 118.6)		80.0 (65.5, 98.0)	93.0 (72.0, 111.5)		34.5 (19.0, 75.8)	45.0 (24.5, 72.0)	
Range	19.0, 175.0	16.0, 161.0		15.0, 145.0	12.0, 162.0		3.0, 150.5	6.0, 173.0	
**Mutation, n (%)**			>0.9			>0.9			<0.001
F508del homozygous	71 (59.2)	131 (58.5)		62 (52.5)	213 (52.1)		143 (47.0)	706 (58.8)	
F508del heterozygous: minimal function	38 (31.7)	78 (34.8)		47 (39.8)	174 (42.5)		88 (28.9)	392 (32.7)	
F508del heterozygous: residual function	3 (2.5)	1 (0.4)		3 (2.5)	9 (2.2)		38 (12.5)	29 (2.4)	
F508del heterozygous: gating	2 (1.7)	1 (0.4)		0 (0.0)	1 (0.2)		23 (7.6)	17 (1.4)	
F508del heterozygous: other	6 (5.0)	13 (5.8)		6 (5.1)	12 (2.9)		12 (3.9)	56 (4.7)	
**Exacerbation at baseline, n (%)**			<0.001			0.2			<0.001
Yes	24 (20.0)	92 (41.1)		29 (24.6)	148 (36.2)		78 (25.7)	529 (44.1)	
No	96 (80.0)	132 (58.9)		89 (75.4)	261 (63.8)		226 (74.3)	671 (55.9)	
Unknown	0 (0.0)	0 (0.0)		0 (0.0)	0 (0.0)		0 (0.0)	0 (0.0)	
**Exacerbation on ETI therapy, n (%)**			>0.9			>0.9			>0.9
Yes	34 (28.3)	64 (28.6)		18 (15.3)	76 (18.6)		71 (23.4)	281 (23.4)	
No	86 (71.7)	160 (71.4)		100 (84.7)	333 (81.4)		233 (76.6)	919 (76.6)	
Unknown	0 (0.0)	0 (0.0)		0 (0.0)	0 (0.0)		0 (0.0)	0 (0.0)	

***Pseudomonas aeruginosa* at baseline, n (%)**			>0.9			0.10			0.069
Yes	16 (13.3)	41 (18.3)		23 (19.5)	134 (32.8)		157 (51.6)	713 (59.4)	
No	104 (86.7)	181 (80.8)		90 (76.3)	265 (64.8)		146 (48.0)	471 (39.3)	
Unknown	0 (0.0)	2 (0.9)		5 (4.2)	10 (2.4)		1 (0.3)	16 (1.3)	
***Pseudomonas aeruginosa* during ETI therapy, n (%)**			>0.9			>0.9			>0.9
Yes	9 (7.5)	21 (9.4)		10 (8.5)	49 (12.0)		114 (37.5)	460 (38.3)	
No	111 (92.5)	203 (90.6)		108 (91.5)	352 (86.1)		190 (62.5)	735 (61.3)	
Unknown	0 (0.0)	0 (0.0)		0 (0.0)	8 (2.0)		0 (0.0)	5 (0.4)	
**CFTR modulator pretreatment, n (%)**	54 (45.0%)	111 (49.6%)	>0.9	45 (38.1%)	142 (34.7%)	>0.9	172 (56.6%)	545 (45.4%)	0.005

[1]Adjusted p-value using the Holm correction for multiple testing.
BMI, body mass index; CFTR, cystic fibrosis transmembrane conductance regulator; ETI, elexacaftor-tezacaftor-ivacftor; IQR, interquartile range; ppFEF25-75, percent predicted mid-expiratory flow; ppFEV1, percent predicted forced expiratory volume in 1 second.
